# Expression and Function of NUMB in Odontogenesis

**DOI:** 10.1155/2013/182965

**Published:** 2013-06-06

**Authors:** Haitao Li, Amsaveni Ramachandran, Qi Gao, Sriram Ravindran, Yiqiang Song, Carla Evans, Anne George

**Affiliations:** ^1^Department of Orthodontics, College of Dental Medicine, Nova Southeastern University, 3200 S. University Drive, Fort Lauderdale, FL 33328, USA; ^2^Brodie Tooth Development Genetics & Regenerative Medicine Research Laboratory, Department of Oral Biology (M/C 690), College of Dentistry, University of Illinois at Chicago, Chicago IL 60612, USA; ^3^Department of Orthdontics, College of Dentistry, University of Illinois at Chicago, 801 S. Paulina Street, Chicago IL 60612, USA

## Abstract

NUMB is a multifunctional protein implicated to function in self-renewal and differentiation of progenitors in several tissues. To characterize the transcripts and to analyze the expression pattern of NUMB in odontogenesis, we isolated 2 full-length clones for NUMB from mouse dental pulp mRNA. One novel sequence contained 200 bp insertion in the phosphotyrosine binding domain (PTB). Confocal microscopy analysis showed strong NUMB expression in human dental pulp stem cells (hDPSC) and preameloblasts. Western blot analysis indicated that NUMB isoforms were differentially expressed in various dental tissues. Immunohistochemical analysis showed that in postnatal mouse tooth germs, NUMB was differentially expressed in the preameloblasts, odontoblasts, cervical loop region, and in the dental pulp stem cells during development. Interestingly, overexpression of NUMB in HAT-7, a preameloblast cell line, had dramatic antagonizing effects on the protein expression level of activated Notch 1. Further analysis of the Notch signaling pathway showed that NUMB significantly downregulates sonic hedgehog (Shh) expression in preameloblasts. Therefore, we propose that NUMB maintains ameloblast progenitor phenotype at the cervical loop by downregulating the activated Notch1 protein and thereby inhibiting the mRNA expression of Shh.

## 1. Introduction

NUMB protein was initially identified in *Drosophila*. Its name comes from the fact that the gene mutation causes flies to lose their sensory neurons and become “numb.” NUMB is demonstrated to play roles in lineage commitment by both gain- and loss-of-functions approaches. Its function has been attributed to asymmetric distribution in daughter cells as well as interaction with Notch signaling components. After its identification, NUMB has been extensively studied in the development of sensory organs, and subsequently in cancer research [1]. We recently identified NUMB as one of the genes that are differentially expressed in rat immortalized odontoblast cells (T4-4) [2]. 

The *Drosophila* NUMB is a membrane-associated protein in the sensory organ precursor cells, and its mutation is lethal resulting from the abnormality of the peripheral nervous systems [3]. Spana et al. identified that the unequal distribution of NUMB in the descendents controls the cell fate of the neurons projecting towards two directions: daughter cells remain as stem cells from maintaining high level NUMB expression, and daughter cells differentiate into neurons with the loss of NUMB expression [4]. NUMB knockout mice die around embryonic day 11.5. They exhibit severe defects in cranial neural tube closure and precocious neuron production, indicating that NUMB promotes progenitor cell fates [5]. Mammalian NUMB is not only expressed in the embryonic tissues but also in most adult tissues, suggesting more complex functions other than neurogenesis [6]. To date, four mammalian NUMB isoforms were identified from alternatively spliced transcripts in neuronal lineage cells [7]. The two members of PRRL isoforms (have long proline rich region) were expressed in early mouse embryonic ages (E7–E10) and were undetectable at E13 when the cells are in rapid expansion stage. The two members of the PRRS isoforms (have short proline rich region) were detected in all developmental stages and adult brains [7, 8]. This differential expression pattern is associated with the function of NUMB in maintaining progenitor cells in the early phase of cortical neurogenesis [9] verses its function in self-renewal and differentiation in late stages of neuron development [10]. Recently, three novel isoforms of NUMB were identified in the human extravillous trophoblast [11].

In rodent incisors, the cervical loop situated at the posterior end of the epithelium consists of progenitor cells which are capable of continuously amplifying and differentiating into amelobasts and support the lifelong growth of the enamel on the labial side [12]. These progenitor cells in the labial cervical loop give rise to the transit-amplifying cells which will further differentiate into preameloblasts and eventually into well-differentiated enamel forming ameloblasts [13]. Notch and sonic hedgehog (Shh) signaling pathway have been shown to be important in regulating this transformation [14].

Seidel et al. demonstrated that Shh produced by the transit amplifying/preameloblasts signals to the stem cells in the cervical loop for continuous ameloblast regeneration. After injecting a hedgehog pathway inhibitor in the mouse mandible, new enamel formation on the labial surface of the mouse incisors was completely blocked. However, the stem cell survival was not endangered due to the fact that normal amelobasts as well as the enamel were able to form after the removal of the hedgehog signal inhibitor [15]. These results clearly revealed the existence of a positive feedback signal loop between the differentiating cells and the stem cells. Shh produced by the differentiating amelobasts signals to the stem cells located in the labial cervical loop to continuously provide progenies in order to support the differentiation demand. In addition, Shh is also important in regulating the preameloblasts to elongate, polarize, and deposit enamel matrix [16]. 

Notch 1, a transmembrane protein, has been shown to be important in specifying dental cell-type identity [14]. Notch signaling regulates the maintenance, fate decision, and proliferation of progenitor cells in multiple tissues such as bone marrow and various neuronal tissues [17]. Notch 1 expressing cells in the cervical loop respond to FGF signaling secreted by the adjacent mesenchyme during mitogenesis and cell fate decision [18]. Inhibition of Notch signaling leads to impaired growth of the mouse incisor cervical loop with reduced proliferation and increased apoptosis. In addition, the inhibition also reduced the population of the ameloblast precursors [19]. 

NUMB inhibits Notch signaling through interaction with the Notch intracellular domain and promotes its ubiquitination and degradation [20]. NUMB was also shown to control the intracellular trafficking of the Notch to suppress its function [21]. Studies also indicated that the expression of NUMB correlates with the suppression of hedgehog signaling [22]. NUMB is also involved in the TP53-activated pathway [23], endocytosis [24], and the determination of cell polarity [25]. These studies suggest that there is a finely tuned regulatory network centered by NUMB in determination of cell fates [26].

Even though the expression of NUMB has been reported in several tissues, there are no reports thus far during tooth development. In this study, we identified several NUMB isoforms that are differently expressed in dental tissues. They were expressed in the cervical loop, immature ameloblasts, odontoblasts, and in specific dental pulp cells. Overexpression of NUMB in preameloblast cells inhibited both the activated Notch 1 protein and Shh expression. Therefore, we propose that NUMB regulates ameloblasts differentiation by modulating the expression levels of Notch 1 protein and Shh expressions. 

## 2. Materials and Methods

### 2.1. RT-PCR

Dental pulp tissues were isolated from postnatal day 5 mouse tooth germs. Single cell suspension was derived by digesting the dental pulp tissues at 37°C for 30 min under constant agitation in a digestion cocktail containing (0.05% trypsin and 0.1% collagenase P). Dental pulp cells were cultured in *α*MEM with addition of 10% fetal bovine serum and 50 U/mL of penicillin and streptomycin for 7 days. RNA was extracted from the cultures using TRIzol reagent (Invitrogen, CA, USA) according to the manufacturer's instructions. cDNA library was generated using SuperScript First-Strand Synthesis System (Invitrogen, CA, USA). Primers were designed to amplify the longest NUMB open reading frame using sequence derived from Ensembl Genome Browser database with the additions of the endonuclease sites on the forward and reverse primers. The primer sequences are Forward: 5′-SalI+ATGAACAAACTACGGC-3′and Reverse: 5′-SacI+TAAAGTTCTATTTCAAAT-3′. PCR products were amplified using high-fidelity Taq with 65°C annealing temperature and 2 minutes extension time.

### 2.2. Immunohistochemistry (IHC)

E13.5, E16.5, E18.5 mouse embryos, postnatal day 3, day 5, and day 7 mouse heads were fixed in 10% neutral buffered formalin for 3 days at 4°C and processed for paraffin section. Serial sections were used for immunohistochemical analysis for NUMB (abcam ab14140) and activated Notch 1 (abcam ab8925) using the Vectastain ABC Kit (Vector Labs, CA, USA) following the manufacturer's instructions. Sections were imaged using Zeiss microscope.

### 2.3. Immunocytochemistry (ICC)

Human dental pulp stem cells (DPSCs), T4-4 preodontoblasts, and HAT-7 cells were plated on sterilized cover slips placed in 6-well culture dishes. Cells were fixed in 10% neutral buffered formalin at 4°C for 2 hours. Cells were permeabilized with 0.5% Triton in PBS for 30 minutes, then blocked in 5% BSA for 1 hour at room temperature, and incubated with primary antibody NUMB (abcam ab14140) and activated Notch 1 (abcam ab8925) overnight at 4°C. TRITC-conjugated goat anti-rabbit secondary antibody and FITC-conjugated goat anti-rabbit secondary antibody were utilized for the visualization of the proteins of interest. Cells were counterstained with nuclei-specific fluorescent stain, DAPI (Vectashield Mounting Medium, CA, USA), and imaged using confocal microscopy with corresponding fluorescent channel. 

### 2.4. Generation of Plasma Membrane Patches

 Plasma membrane patches from HAT-7 cells were prepared as described previously [27, 28]. The patches were fixed in 4% paraformaldehyde and immunostained with anti-NUMB antibody.

### 2.5. Total Protein Lysate

Total protein lysate was prepared using RIPA buffer (50 mM Tris, 150 mM NaCl, 0.1% SDS, 0.5% Na. Deoxycholate, and 1% NP40) in the presence of proteinase inhibitor cocktail (Sigma, MO, USA). For protein extraction from cell lines, the cells were cultured in 100 mm petri dish and washed with ice-cold PBS at the time of harvest. Cells were lysed with 700 *μ*L of RIPA buffer, scraped off from the culture dish, vortexed for 10 seconds, and incubated on ice for 10 minutes. The supernatant was collected after centrifugation at 14 k rpm at 4°C. For protein extraction from whole tooth buds, day 4 postnatal mouse tooth buds were dissected under the microscope. The tooth buds were frozen with liquid nitrogen, ground in a mortar and pestle with the addition of the RIPA buffer supplemented with proteinase inhibitor cocktail, and processed as above. 

### 2.6. Western Blot Analysis

 Total protein extracted from cell cultures and embryonic tooth germs was quantified using Bradford protein assay with an MBA 2000 spectrometer (Perkin Elmer, MA, USA). A total of 35–40 *μ*g of protein were loaded on the 10% SDS polyacrylamide gel for separation. Well-separated proteins were transferred onto a nitrocellulose membrane at 4°C. The nitrocellulose membrane was then blocked with 5% nonfat milk in PBS for 1 hour and then incubated with primary antibody in 3% BSA/PBS overnight (NUMB: abcam ab14140; 1/1500 dilution; activated Notch 1: abcam; ab8925 1/1600; tubulin 1/3000) under constant agitation at 4°C. The blot was incubated with the secondary antibody (goat antirabbit conjugated with HRP 1/3000) at room temperature for 1 hour. Chemiluminescent Western Blot Substrate (Thermo Scientific Pierce ECL, IL, USA) was utilized for developing, and the blot was exposed on CL-XPosure Film (Thermo Scientific, IL, USA).

### 2.7. Culturing HAT-7 Cells

HAT-7 cells [29] were maintained in DMEM/F-12 medium with addition of 10% FBS and 50 U/mL of penicillin and streptomycin. To overexpress NUMB, HAT-7 cells were plated in 6-well culture dish 24 hour in advance with 90% confluence in antibiotic-free medium. Plasmids including pSR-GFP/Neo (gifts from Wang et al. [30]); GFP-NUMB (NUMB isoform 4) (gifts from Nishimura and Kaibuchi [31]) were reconstituted in serum-free and antibiotics-free DMEM/F-12 medium for transfection by Lipofectamine 2000 (Invitrogen). Transfection medium was replaced with fresh culture medium in 24 hours. Cells were allowed to recover for 48 hours before being placed in selection medium. The selection medium contains 1 to 100 dilution of the G418 neomycin sulfate (Sigma) stock solution (100 mg/mL) in DMEM/F-12 medium. The G418 concentration was determined from the killing curve where 50% of the cells were killed in 48 hours. Cells were cultured in selection medium for 6 weeks before any experiments were carried out in order to completely remove the nontransfected or transiently transfected cells. Cells were maintained in the selection medium hereafter. For the PCR-based Notch 1 superarray signaling pathway analysis, transiently transfected cells for 48 hours were utilized. 

### 2.8. Functional Analysis of NUMB in Notch Signaling Using Pathway-Specific PCR-Based Superarray

HAT-7 cells were transiently transfected with NUMB-GFP expression vector and control Neomycin-GFP expression vector. RNA was extracted using Qiagen RNA easy kit following the manufacturer's protocol. cDNA was generated using RT First-Strand cDNA Synthesis Kit (C-03 Qiagen, CA, USA) from 4 *μ*g of RNA. Genomic DNA was eliminated by on column DNase digestion during RNA extraction and before reverse transcription. RT² Profiler PCR Array System was used for analyzing the mouse Notch 1 signaling pathway (PAMM-059C). 

## 3. Results

### 3.1. Expression of NUMB Transcripts in Dental Tissue

**Figure 1:**
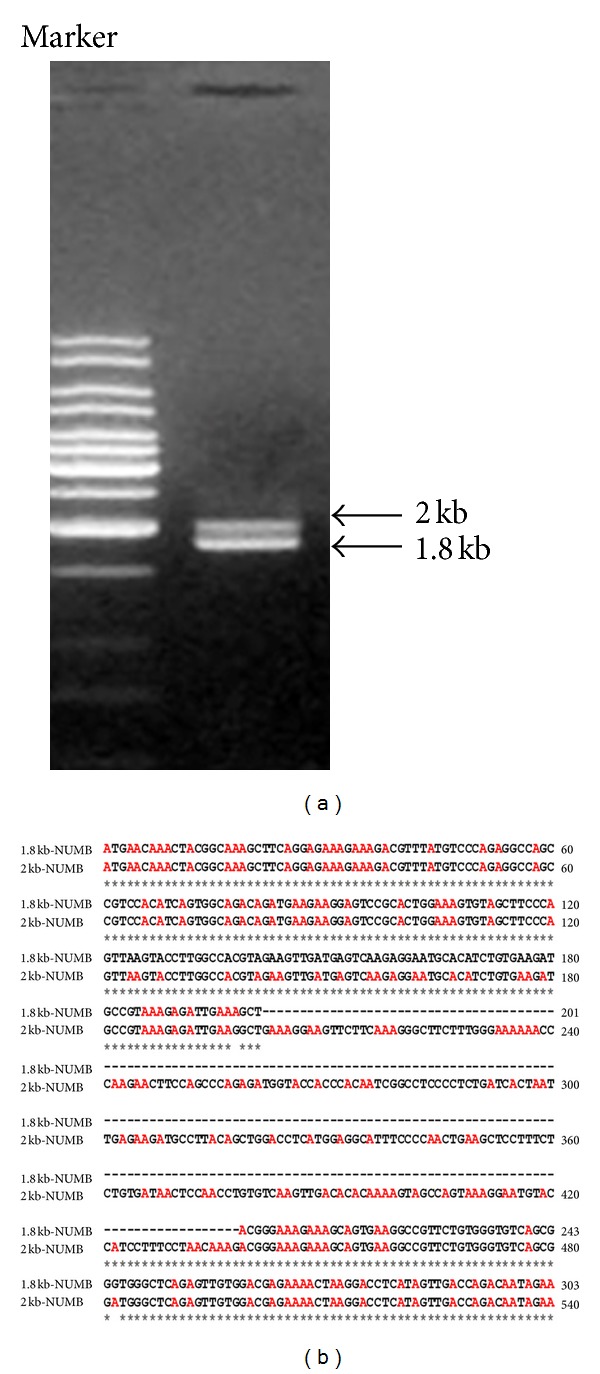
(a) RT-PCR analysis of NUMB isoforms in day 7 dental pulp cells. RT-PCR analysis of mRNA extracted from day 7 dental pulp culture shows two PCR bands corresponding to 2 kb and 1.8 kb obtained using primers designed to amplify the longest NUMB reading frame. (b) The alignment of the sequence obtained from 1.8 kb and the 2 kb PCR fragments. The 2 kb PCR fragment contains 236 bp insertion in the phosphotyrosine binding domain (PTB) 201 bp downstream of the initiation start site.

To demonstrate the presence of NUMB transcripts in dental pulp cells, we performed RT-PCR using cDNA generated from day 7 primary dental pulp cultures. Primers were designed to amplify the longest NUMB open reading frame. Two specific PCR fragments were amplified (Figure 1(a)). Sequence analysis indicated that the 1.8 kb PCR fragment is consistent with the mRNA sequence of NUMB isoform 4. The 2 kb PCR fragment contains 236 bp insertion in the phosphotyrosine binding domain (PTB) 201 bp downstream of the initiation codon (Figure 1(b)). However, protein prediction analysis from the 2 kb DNA fragment did not lead to a longer protein product but a protein of 100 amino acids that contained an early termination codon. 

### 3.2. Expression of NUMB in Dental Cell Lines

**Figure 2:**
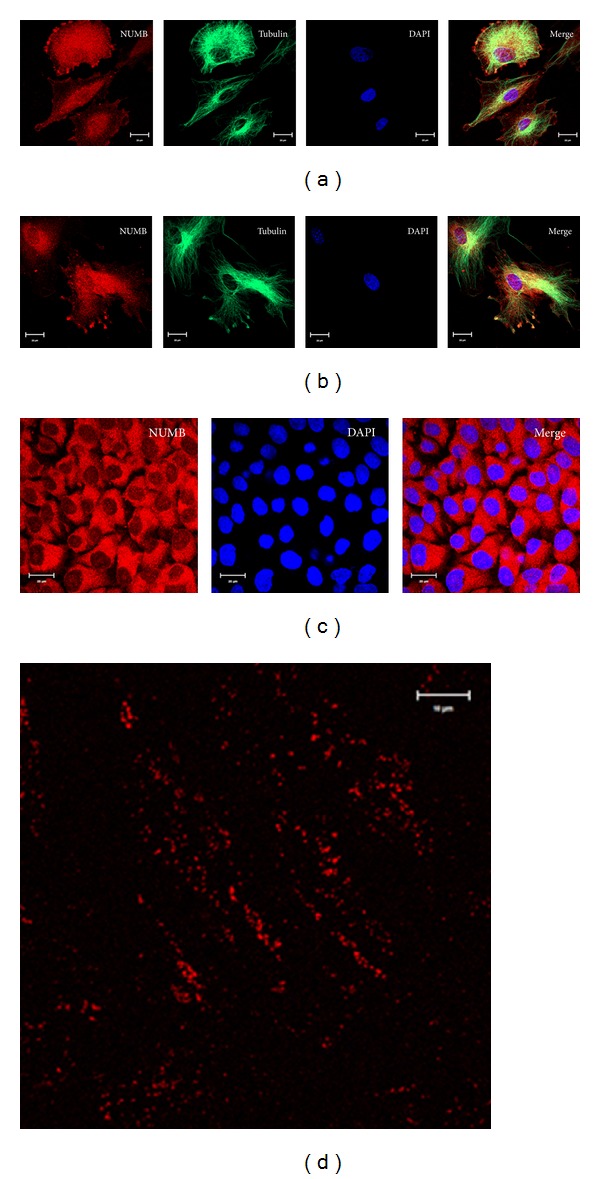
Localization of NUMB in various odontogenic cells: immunocytochemistry staining of NUMB expression in T4-4 odontoblast cells (a), human dental pulp stem cell (b), and preameloblast cells (c). Membrane patches isolated from preameloblast HAT-7 cells stained using NUMB antibody (d).

To investigate the subcellular localization of NUMB, immunocytochemical analysis was performed on human dental pulp stem cells (DPSCs), T4-4 preodontoblasts, and HAT-7 preameloblast cells (Figure 2). NUMB was clearly expressed in these 3 cell types. Specifically, NUMB was localized on the cell membrane, within the cytoplasm, and in the nucleus in only the DPSCs. In order to confirm the localization of NUMB on the cell membrane, plasma membrane patches from HAT-7 cells were used in immunostaining. The positively stained cell membrane patches by NUMB antibody confirmed the presence of NUMB on the cell membrane of HAT-7 cells (Figure 2(d)). 

**Figure 3:**
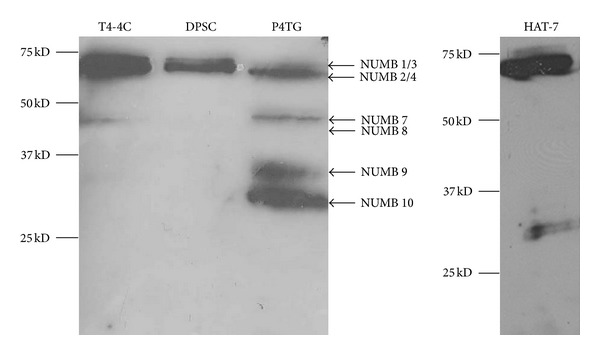
Western blot analysis of NUMB isoforms in odontogenic cells: total protein lysates were obtained from odontoblasts (T4-4 cells); dental pulp stem cells (DPCs); preameloblasts (HAT-7); tooth germs isolated from postnatal day 4 molars (P4TG). Notice that NUMB isoforms are differentially expressed in these tissues.

In order to identify the NUMB isoforms that are present in the dental tissue, Western blot analysis was performed using total protein lysates prepared from postnatal day 4 tooth germ and from the dental cell lines (Figure 3). All the high molecular weight NUMB proteins ~72 KD and ~66 KDa were detected in all the dental tissues (proteins that have 1 KD difference were unable to be separated). To our surprise, multiple low molecular weight proteins located between 25 KD and 50 KD were also detected in the tooth germ as well as in HAT-7 cells. Since there are no published reports on the proteolytic cleavage of NUMB, we speculated that these could be due to NUMB isoforms expressed in dental tissues that have not yet been identified. While this work was in progress, two new isoforms, namely, NUMB 5 and NUMB 6 were published [32]. These proteins were described to be rare and were transiently expressed in cancer cells. The novel isoforms identified by Karaczyn et al. [32] appeared to have higher molecular weight than the proteins we identified. For the sake of simplicity, in Figure 3, we have described the 37–50 KD proteins as NUMB 7 and NUMB 8 and the 25–37 KD proteins as NUMB 9 and NUMB10. This needs to be further validated by mass spectrometry or protein sequencing analysis and determine if it is similar to the NUMB 7 and 8 isoform reported by Haider et al. in human placenta [11]. The ameloblast cell line, HAT-7 cells, expresses high levels of NUMB 1/2 and NUMB 3/4, as well as NUMB 9/10 but is deficient in the expression of NUMB 7/8. The dental pulp cells show very low levels of low molecular weight NUMB isoform expression. Among the high molecular weight NUMB isoforms, the NUMB 2/4 appeared to be the major isoforms in the dental tissue. These data suggest that there is differential expression of NUMB isoforms in dental tissues. 

### 3.3. Expression of NUMB in Developing Tooth Germs

**Figure 4:**
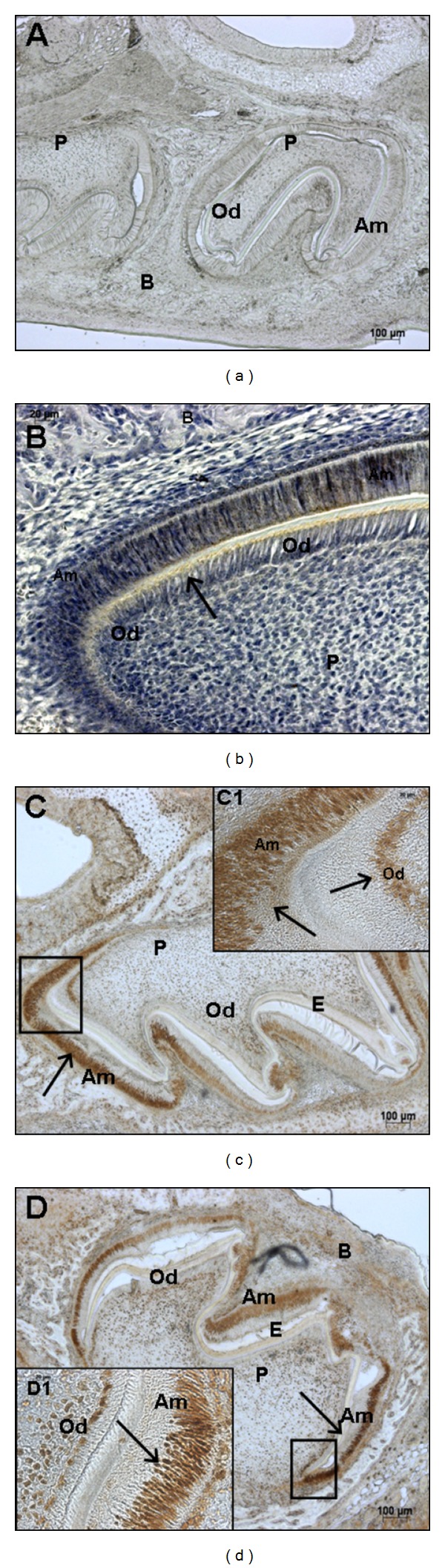
Localization of NUMB in odontogenic tissues at postnatal day 3: localization of NUMB in P3 incisors (B) and molars (C and D). NUMB can be detected in the ameloblasts (Am); odontoblasts (Od); ameloblast progenitors (cervical loop); in the dental pulp cells (P) in close vicinity to the odontoblasts. (D1) shows the immature ameloblasts, immature odontoblasts, and dental pulp cells from incisor and molar, respectively. (a) is the negative control. Scale bar represents 100 *μ*m for (a), (c), and (d) and 20 *μ*m for C1 and D1, respectively.

**Figure 5:**
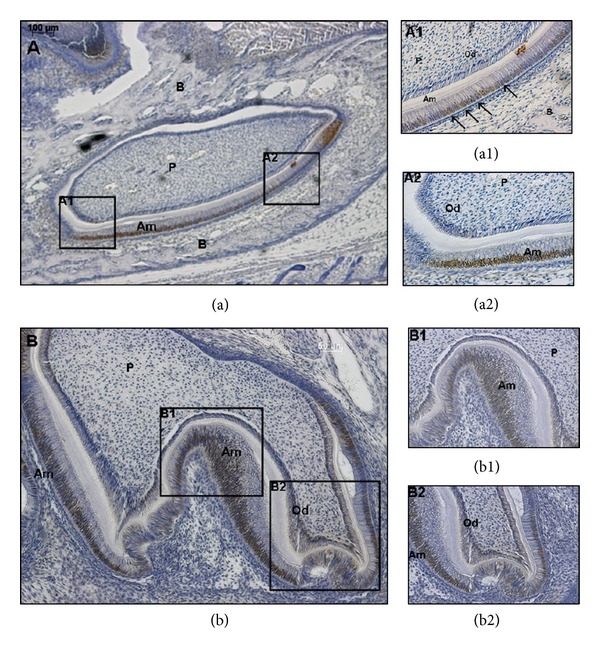
Localization of NUMB in odontogenic tissues at postnatal day 5: NUMB expression can be detected in the ameloblasts and odontoblasts. Notice that in (a1), NUMB is positively stained in some ameloblasts but negatively stained in the adjacent cells (indicated by arrows). Scale bar represents 100 *μ*m for (a); 50 *μ*m for (b); 20 *μ*m for (a1), (a2), and (b1), (b2), respectively.

**Figure 6:**
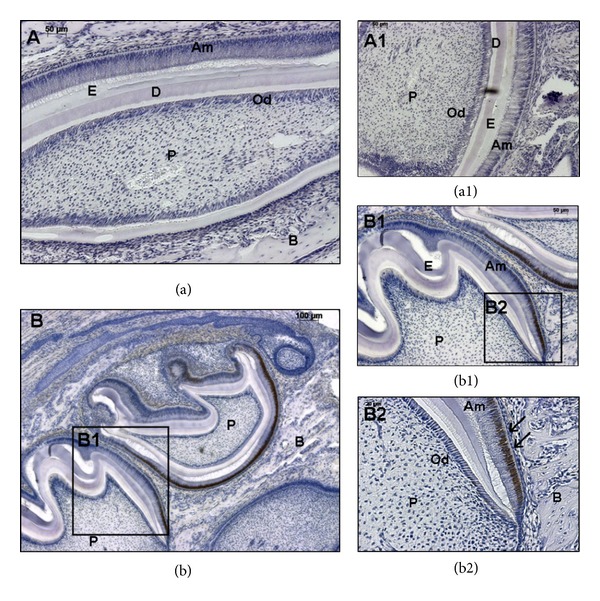
Localization of NUMB in odontogenic tissues at postnatal day 7: NUMB expression is more defined in the immature ameloblasts, and very low expression levels can be detected in the immature odontoblasts. Scale bar represents 50 *μ*m for (a), 100 *μ*m for (b), and 50 and 20 *μ*m for (a1), (b1), and (b2), respectively.

NUMB expression patterns were analyzed in the developing tooth germs from embryonic stage 13.5 to postnatal day 7 by immunohistochemistry. At E13.5, E16.5, and E18.5, there was no detectable NUMB expression in the developing tooth germs (data not shown). However, during postnatal tooth development, there was strong NUMB expression. Mouse incisors have enamel and dentin present on the labial surface, and only dentin on the lingual surface [33]. In P3 mouse incisor, NUMB protein was strongly expressed in the ameloblasts on the labial surface, in the cervical loop, in the odontoblasts at both labial and lingual surfaces, and in the dental pulp cells in close vicinity to the odontoblasts. In the P3 molar, NUMB expression can be detected in the ameloblasts, immature odontoblasts, and the dental pulp cells adjacent to the odontoblasts (Figure 4). In the P5 incisor, NUMB is localized in the ameloblasts and immature odontoblasts, and the expression is less intense compared to the P3 dental tissues. Also, there was no expression of NUMB in the dental pulp cells (Figure 5). In P7 dental tissues, the NUMB expression is more specifically limited to the stem cells of the stratum intermedium (Figure 6). Overall, in the postnatal dental tissues, NUMB is expressed in the ameloblast progenitors in the cervical loop of the incisor, immature ameloblasts and odontoblasts, and in the dental pulp cells in the vicinity of the odontoblasts. Note that some ameloblasts in P5 and P7 express NUMB, but their neighboring cells do not, indicating that NUMB is asymmetrically distributed in the daughter cells. 

### 3.4. NUMB Downregulates Activated Notch 1

The *in vivo* temporal and spatial expression pattern of NUMB in the developing tooth germ suggested that NUMB may play a role in regulating ameloblast differentiation. Our study shows that NUMB has a defined expression pattern in the ameloblast lineage cells, more specifically in the ameloblast progenitors and preamelobasts. Several publications have reported that Notch signaling is critical in ameloblast differentiation, and NUMB is involved in regulating Notch1 proteolysis. Therefore, we investigated the relationship of NUMB and Notch 1 in preameloblasts. 

**Figure 7:**
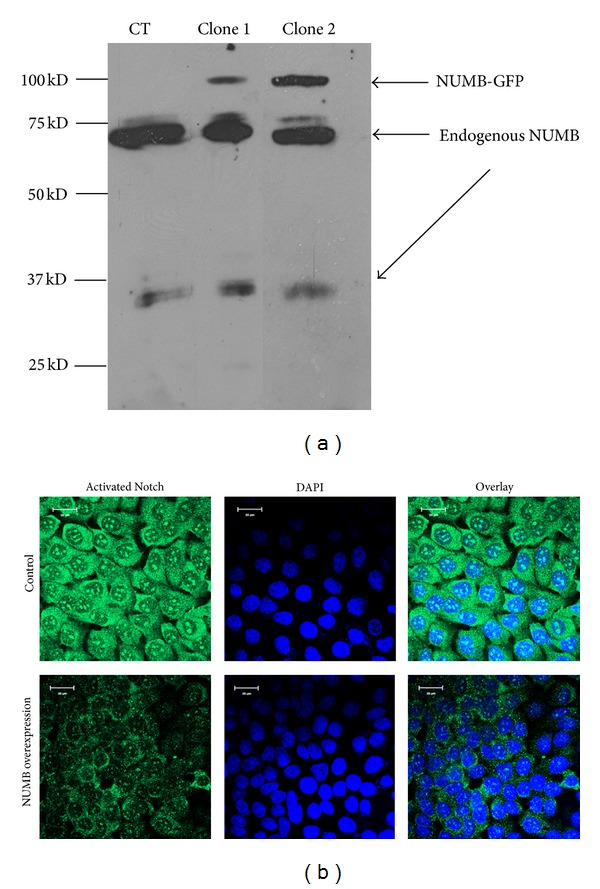
Overexpression of NUMB-GFP in HAT-7 cells: (a) Western blot analysis to detect overexpression of NUMB-GFP in HAT-7 cells. Total proteins were isolated from 2 clones, namely, clone 1 and 2. (b) Immunohistochemical analysis detected that Notch1 protein expression is dramatically reduced in the NUMB overexpressing cell line (stable cell line clone 2 was used). The green fluorescence represents activated Notch1 protein.

We first established that NUMB and Notch 1 colocalized in the same cell types. Activated Notch 1 was found to be strongly expressed in ameloblasts, odontoblasts, and dental pulp cells (see Supplementary Data 1 available online at http://dx.doi.org/10.1155/2013/182965). In order to study the functional relationship between NUMB and activated Notch 1, we generated NUMB overexpressing HAT-7 cells (Figure 7(a)). The activated Notch 1 expression pattern was evaluated by immunocytochemical analysis. Confocal microscopy clearly indicated that the NUMB overexpressing HAT-7 cells had dramatically reduced expression of activated Notch 1 represented by the expression of green fluorescence (Figure 7(b)). 

**Figure 8:**
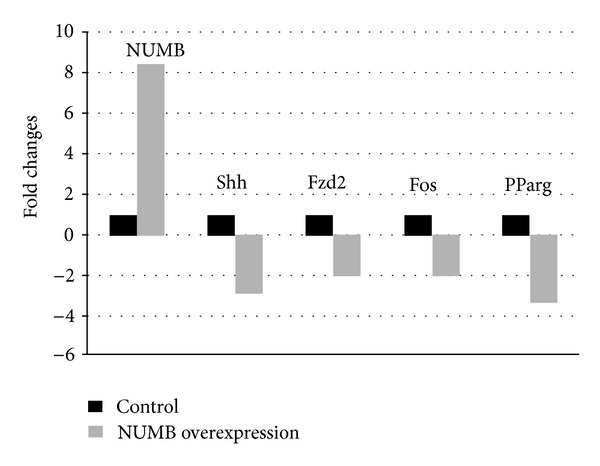
Notch signaling pathway PCR array. Genes from the Notch1 signaling pathway that were affected by NUMB overexpression.

**Figure 9:**
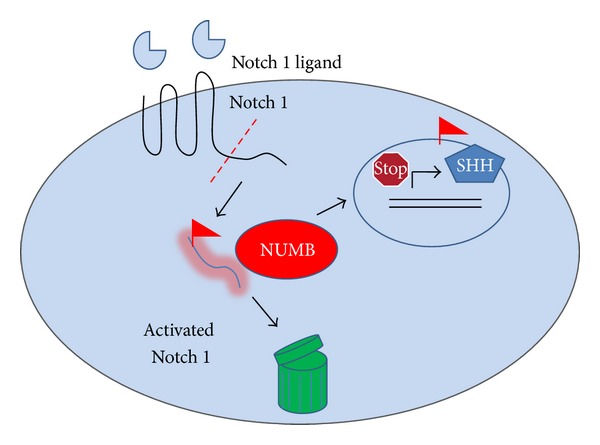
Hypothetical model. NUMB regulates stem cell properties at two levels. NUMB downregulates the expression of the activated Notch 1 protein level in the progenitor cells at the cervical loop and thereby inhibits the Shh expression in the adjacent preameloblasts. Thus, NUMB influences the expression of downstream genes such as Notch1 and Shh and thus performs critical role in ameloblast differentiation.

Next, we investigated the key players in the Notch 1 signaling pathway using a PCR-based array. The RT² Profiler PCR Array profiles the expression of 84 genes involved in Notch signaling. Results in Figure 9 show that NUMB mRNA is 7.5 times upregulated in the NUMB overexpressing cells compared to the control vector transfected cells. Majority of the genes in the Notch 1 signaling pathway were down-regulated. Noteworthy among them were Shh, Fos, Fzd2, and PParg with *P* values < 0.05 (Figure 8).

## 4. Discussion

While we were isolating the NUMB isoforms expressed in dental pulp cells using primers designed to amplify the longest reading frame, we repeatedly obtained two PCR fragments. The 1.8 kb fragment is consistent with published NUMB isoform 4 cDNA sequence, while the 2 kb PCR fragment contained a 237 bp insertion in the PTB domain. The predicted protein sequence indicated an early termination codon which allows only 103 amino acids being translated. The NUMB antibody utilized in this study was raised against the NUMB C-terminal polypeptide (the synthetic peptide derived from residues 600 to the C-terminus of human NUMB). Therefore, isoforms that end upstream of this polypeptide were not detected. The total protein lysate from the whole tooth germ contained all the detectable NUMB isoforms. The high molecular weight NUMB isoform which is the major published isoform is expressed in all dental tissues. 40 KDa isoform was detected in the odontoblasts, and the low molecular weight NUMB isoform (30 KDa) was predominantly expressed in the ameloblasts. While this paper was in preparation, 2 new NUMB isoforms, namely, NUMB 5 and 6 were reported and identified in the human cancer cells [32]. These two new isoforms are reported to be above 50 KD with 10 KD difference. They were characterized as being expressed rarely and transiently in these cancer cells. Recently, NUMB 7, 8, and 9 isoforms were identified by RT-PCR cloning in the human extravillous trophoblast [11]. In this study, four possible NUMB isoforms were detected in dental tissues. Further analysis is required to confirm the protein sequences. The differentially expressed NUMB isoforms in various dental tissues and possibly at different stages of tooth development suggest that NUMB might play specific functional role during tooth development. 

Subcellular localization of NUMB in various odontogenic cell lines showed its localization in the cytoplasm, nucleus, and the cell membrane. Nuclear NUMB is particularly interesting, and its functional role in the nucleus has yet to be determined. Immunohistochemical analysis showed prominent NUMB expression during early tooth development. No NUMB expression was detected during embryonic development of the tooth. Strong NUMB expression was detected in the early postnatal development and was mainly localized in immature ameloblasts, preamelobasts, cervical loop, immature odontoblasts, and dental pulp cells. 

As NUMB is regulated by Notch 1, we therefore examined the effect of overexpression of NUMB on Notch expression in preameloblasts. Results from this study showed that overexpression of NUMB dramatically reduced the expression of activated Notch 1 protein expression in HAT-7 cells. Further, we examined if the reduced expression of Notch1 had an effect on downstream signaling events. Results from this study showed that with NUMB overexpression (7.5-fold increase), several genes in the Notch signaling pathway were downregulated. Most notable was the downregulation of sonic hedgehog. 

The preameloblast HAT-7 cells used in this study were derived from the rat incisor cervical loop and its proximity [29]. This region contains stem cell population as well as immature preameloblasts. Based on our findings, we suggest that NUMB can have multiple effects on ameloblast differentiation by exerting its effect on both the stem cells and the preameloblasts. In the stem cells at the cervical loop, NUMB is highly expressed, and this downregulates Notch 1 which in turn inhibits Shh mRNA expression. Thus, the expression of NUMB is responsible for the maintenance of the progenitor cells in the cervical loop. During development, NUMB regulates the differentiation of preameloblasts into ameloblasts. In day 3, NUMB is expressed in the preameloblasts, but by day 7 there is less NUMB expression and thereby relieving its influence on suppressing the expression of Shh. This differential expression of NUMB and Shh in preameloblasts might be necessary for its differentiation and polarization. This observation corroborates well with published reports that demonstrate the critical role that NUMB plays in regulating cell polarization and differentiation [23–25]. 

 Based on our findings, we propose a hypothetical model as shown in Figure 9. NUMB regulates the putative stem cell number and property by inhibiting Notch 1 signaling and thereby downregulating the expression of Shh. At the same time, NUMB can influence the differentiation of preameloblasts to ameloblasts. The signaling mechanism involved in this differentiation cascade will be pursued in the future.

## Supplementary Material

Supplementary figure 1: E16.5 mouse tooth germ was sectioned and immunostained with NUMB antibody. No NUMB expression was detected in these tooth germs. The right panel shows a higher resolution of the tooth germ.
Supplementary figure 2: Postnatal day 5 incisors were sectioned and immunostained for NUMB and activated Notch 1 antibodies, Activated Notch 1 expression was detected in ameloblasts, odontoblasts and dental pulp cells. Our results indicates both activated Notch 1 protein and NUMB protein are expressed in ameloblasts.
