# Supplementary material for: Expression and Function of NUMB in Odontogenesis
Source: Biomed Res Int. 2013 Jun 6;2013:182965. doi: 10.1155/2013/182965 (PMC3690219; doi:10.1155/2013/182965)
Supplement: Supplementary file 1 — Supplementary figure 1: E16.5 mouse tooth germ was sectioned and immunostained with NUMB antibody. No NUMB expression was detected in these tooth germs. The right panel shows a higher resolution of the tooth germ. Supplementary figure 2: Postnatal day 5 incisors were sectioned and immunostained for NUMB and activated Notch 1 antibodies, Activated Notch 1 expression was detected in ameloblasts, odontoblasts and dental pulp cells. Our results indicates both activated Notch 1 protein and NUMB protein are expressed in ameloblasts. [file 182965.f1.pdf]

Supplementary data 1

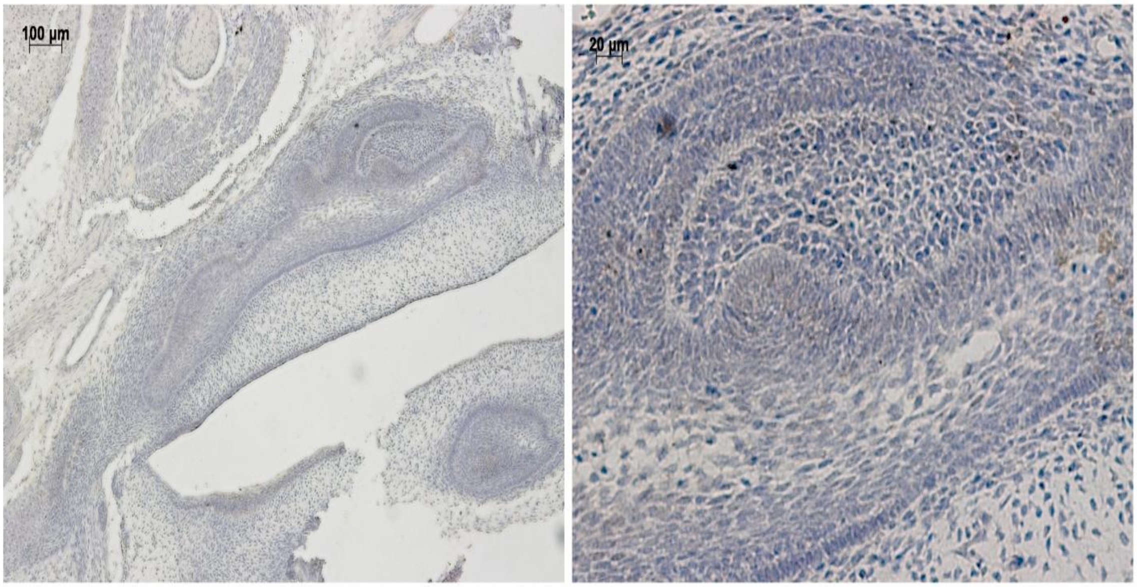

Supplementary data 1: E16.5 mouse tooth germ was sectioned and immunostained with NUMB antibody. No NUMB expression was detected in these tooth germs. The right panel shows a higher resolution of the tooth germ.

Supplementary data 2

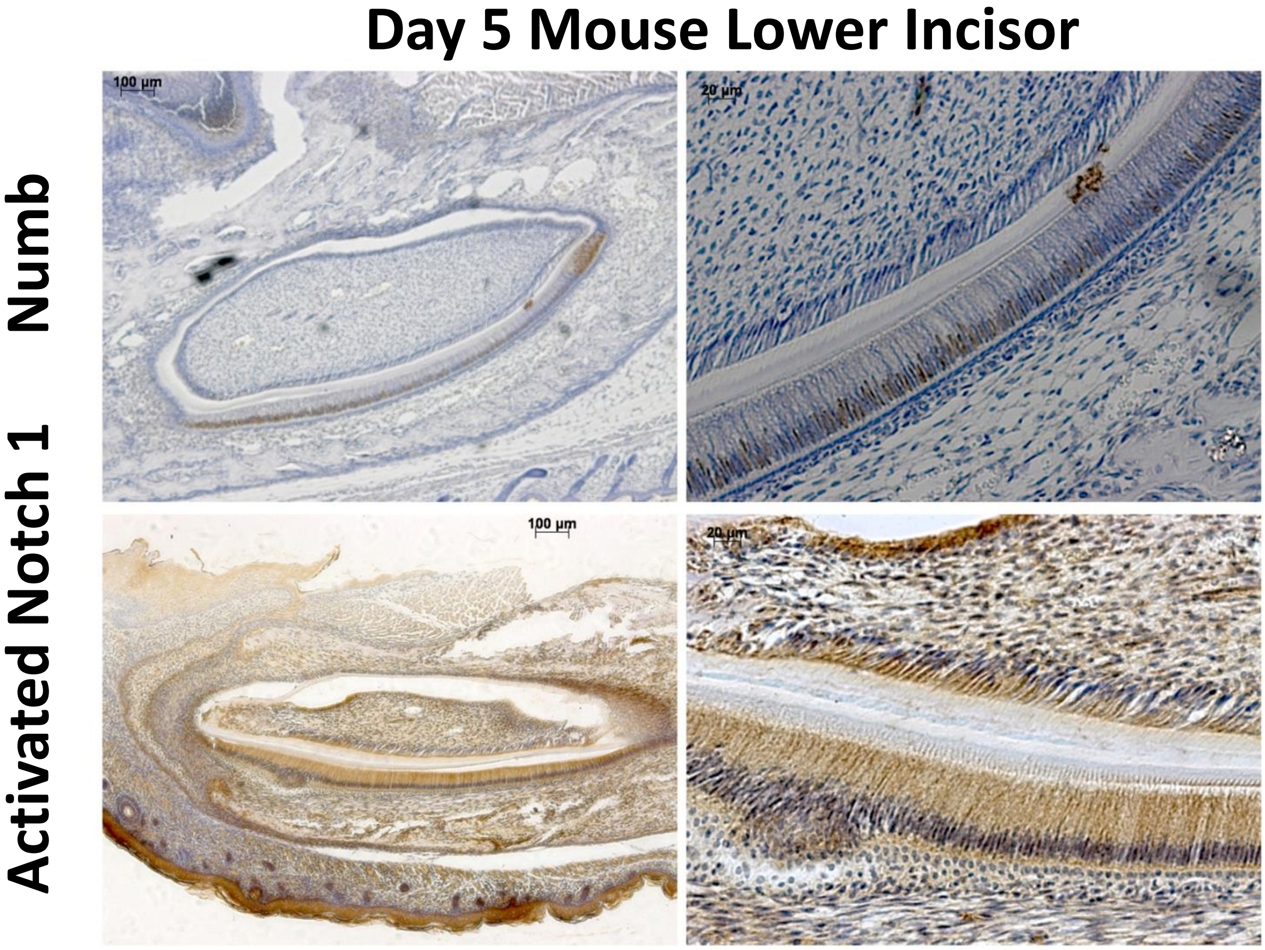

Supplementary data 2: Postnatal day 5 incisors were sectioned and immunostained for NUMB and activated Notch 1 antibodies, Activated Notch 1 expression was detected in ameloblasts, odontoblasts and dental pulp cells. Our results indicates both activated Notch 1 protein and NUMB protein are expressed in ameloblasts.
